# GC-MS analysis of insecticidal essential oil of flowering aerial parts of *Saussurea nivea* Turcz

**DOI:** 10.1186/2008-2231-20-14

**Published:** 2012-08-28

**Authors:** Sha Sha Chu, Guo Hua Jiang, Zhi Long Liu

**Affiliations:** 1 Department of Entomology, China Agricultural University, Haidian District, Beijing, 100193, China; 2 Analytic and Testing Center, Beijing Normal University, Haidian District, Beijing, 100875, China

**Keywords:** *Saussurea nivea*, *Sitophilus zeamais*, Contact toxicity, Fumigant, Essential oil composition

## Abstract

**Background:**

Several species from *Saussurea* have been used in the traditional medicine, such as *S. lappa*, *S. involucrate,* and *S. obvallata*. There is no report on medicinal use of *S. nivea*. The aim of this research was to determine chemical composition and insecticidal activity of the essential oil of *S. nivea* Turcz (Asteraceae) aerial parts against maize weevils (*Sitophilus zeamais* Motschulsky) for the first time.

**Results:**

Essential oil of *S. nivea* flowering aerial parts was obtained by hydrodistillation and analyzed by gas chromatography–mass spectrometry (GC-MS). A total of 43 components of the essential oil of *S. nivea* were identified. The principal compounds in the essential oil were (+)-limonene (15.46%), caryophyllene oxide (7.62%), linalool (7.20%), α-pinene (6.43%), β-pinene (5.66%) and spathulenol (5.02%) followed by β-eudesmoll (4.64%) and eudesma-4,11-dien-2-ol (3.76%). The essential oil of *S. nivea* exhibited strong contact toxicity against *S. zeamais* with an LD_50_ value of 10.56 μg/adult. The essential oil also possessed fumigant toxicity against *S. zeamais* with an LC_50_ value of 8.89 mg/L.

**Conclusion:**

The study indicates that the essential oil of *S. nivea* flowering aerial parts has a potential for development into a natural insecticide/fumigant for control of insects in stored grains.

## Background

The maize weevil (*Sitophilus zeamais* Motschulsky) is one of the major pests of stored grains and grain products in the tropics and subtropics [1]. Infestations not only cause significant losses due to the consumption of grains; they also result in elevated temperature and moisture conditions that lead to an accelerated growth of molds, including toxigenic species [2]. Currently, control of stored product insects relies heavily on the use of synthetic insecticides and fumigants, which has led to problems such as disturbance of the environment, increasing application costs, pest resurgence, pest resistance to pesticides and lethal effects on non-target organisms in addition to direct toxicity to the users [3]. Thus, there is a considerable interest in developing natural products that are relatively less damaging to mammalian health and the environment than existing conventional pesticides, as alternatives to non-selective synthetic pesticides to control the pests of medical and economic importance [4,5]. In recent years, various workers have been concentrating their efforts on the search for natural products as an alternative to conventional insecticides and fumigants, as well as the re-evaluation of traditional botanical pest control agents [5]. Essential oils or their constituents may provide an alternative to currently used fumigants/pesticides to control stored-food insects. Investigations in several countries confirm that some plant essential oils not only repel insects, but possess contact and fumigant toxicity against stored product pests as well as exhibited feeding inhibition or harmful effects on the reproductive system of insects [5,6]. In addition, it has been shown that essential oils have antibacterial and antinematicidal activities [7-14].

During the screening program for new agrochemicals from Chinese medicinal herbs and wild plants, the essential oil of *Saussurea nivea* Turcz (synonym: *Himalaiella nivea*; *Aplotaxis nivea*; *Saussurea deltoidea var. nivea*; and *Saussurea crispa*) [15] flowering aerial parts was found to possess strong insecticidal toxicity against the grain storage insect, *S. zeamais*. *Saussurea* is a genus of about 300 species of flowering plants in the family Asteraceae, native to cool temperate and arctic regions of Asia, Europe, and North America. Many species of *Saussurea* were used in traditional medicine such as *S. lappaS. involucrate,* and *S. obvallata*. For example, *S. involucrata* aerial parts have long been used in traditional Chinese medicine for the treatment of rheumatoid arthritis, cough with cold, stomachache, dysmenorrhea, and altitude sickness, and have antiinflammatory, cardiotonic, abortifacient, anticancer, and antifatigue actions [16]. However, there is no report on medicinal use of *S. nivea*. *S. nivea* is an herbaceous perennial plant distributed mainly in the north of China (Beijing, Hebei, Liaoning, Gansu, Ningxia, Shaanxi, Shanxi Province and Inner Mongolia) and Korea [16]. The aqueous extract of this plant was used to control insect pests by the local farmer [17]. Five constituent compounds (quercetin-3-*O*-β-*D*-glucoside, kaempferol-3-*O*-β-*D*-glucoside, α-amyrin, β-sitosterol, hentiantane) have been isolated from the ethanol extract of *S. nivea*[17]. However, a literature survey has shown that there is no report on the volatile constituents and insecticidal activity of *S. nivea*; thus we decided to investigate the chemical constituents and insecticidal activities of the essential oil of *S. nivea* aerial parts against grain storage insect for the first time.

## Materials and methods

### Plant material

The aerial parts of *S. nivea* at flowering state were collected in August 2009 from Xiaolongmen National Forest Park (39.48° N latitude and 115.25° E longitude, Mentougou District, Beijing 102300). The sample was air-dried and identified by Dr. Liu, Q.R. (College of Life Sciences, Beijing Normal University, Beijing 100875, China) and a voucher specimen (ENTCAU-Compositae-10014) was deposited at the Department of Entomology, China Agricultural University (Beijing 100193). The sample was ground to a powder using a grinding mill (Retsch Mühle, Germany). Each 600 g portion of powder was mixed in 1,800 ml of distilled water and soaked for 3 h. The mixture was then boiled in a round-bottom flask, and steam distilled for 6–8 h. Volatile essential oil from distillation was collected in a flask. Separation of the essential oil from the aqueous layer was done in a separatory funnel, using *n*-hexane. The solvent was evaporated using rotary evaporator (BUCHI Rotavapor R-124, Switzerland). The sample was dried over anhydrous Na_2_SO_4_ and kept in a refrigerator (4°C) for subsequent experiments.

### Insects

The maize weevils (*S. zeamais*) were obtained from laboratory cultures in the dark in incubators at 29-30 °C and 70-80% relative humidity and were reared on whole wheat at 12-13% moisture content in glass jars (diameter 85 mm, height 130 mm). Unsexed adult weevils used in all the experiments were about one week old. All containers housing insects and the petri dishes used in experiments were made escape proof with a coating of polytetrafluoroethylene (Fluon, Blades Biological, UK).

### Gas chromatography–mass spectrometry

The essential oil of *S. nivea* was subjected to GC-MS analysis on an Agilent system consisting of a model 6890 N gas chromatograph, a model 5973 N mass selective detector (EIMS, electron energy, 70 eV), and an Agilent ChemStation data system. The GC column was an HP-5 ms fused silica capillary with a 5% phenyl-methylpolysiloxane stationary phase, film thickness of 0.25 μm, a length of 30 m, and an internal diameter of 0.25 mm. The GC settings were as follows: the initial oven temperature was held at 60 °C for 1 min and then heated at 180 °C at a rate of 10 °C/min, held for 1 min, and then heated to 280 °C at 20 °C/min and held for 15 min. The injector temperature was maintained at 270 °C. The sample (1 μl) was injected neat, with a split ratio of 1: 10. The carrier gas was helium at flow rate of 1.0 mL min^−1^. Spectra were scanned from 20 to 550 m/z at 2 scans s^−1^. Most constituents were identified by gas chromatography by comparison of their retention indices with those of the literature or with those of authentic compounds available in our laboratories. The retention indices were determined in relation to a homologous series of *n*-alkanes (C_8_–C_24_) under the same operating conditions. Further identification was made by comparison of their mass spectra with those stored in NIST 08 and Wiley 275 libraries or with mass spectra from literature [18]. Component relative percentages were calculated based on normalization method without using correction factors.

### Contact toxicity by topical application

Range-finding studies were run to determine the appropriate testing concentrations of the essential oil of *S. nivea*. A serial dilution of the essential oil (5.0%-15.0%, 5 concentrations) was prepared in *n*-hexane. Aliquots of 0.5 μl per insect were topically applied dorsally to the thorax of the weevils, using a Burkard Arnold microapplicator. Controls were determined using 0.5 μl *n*-hexane per insect. Ten insects were used for each concentration and control, and the experiment was replicated six times. Both the treated and control weevils were then transferred to glass vials (10 insects/vial) with culture media and kept in incubators at 29-30°C and 70-80% relative humidity. Mortality was observed after 24 h. The insects were considered dead if appendages did not move when probed with a camel brush. The observed mortality data were corrected for control mortality using Abbott’s formula. Results from all replicates were subjected to probit analysis using the PriProbit Program V1.6.3 to determine LD_50_ values [19].

### Fumigant toxicity bioassay

Range-finding studies were run to determine the appropriate testing concentrations of *S. nivea* essential oil. The fumigant toxicity of *S. nivea* essential oil was determined by used the method of Liu and Ho [1] with some modifications. A Whatman filter paper (diameter 2.0 cm) was placed on the underside of the screw cap of a glass vial (diameter 2.5 cm, height 5.5 cm, volume 24 ml). Ten microliters of the essential oil (5.39-20.00%, 6 concentrations) was added to the filter paper. The solvent was allowed to evaporate for 15 s before the cap was placed tightly on the glass vial (with 10 unsexed insects) to form a sealed chamber. The vials were upright and the Fluon (ICI America Inc) coating restricted the insects to the lower portion of the vial to prevent them from the treated filter paper. They were incubated at 27-29°C and 70-80% relative humidity for 24 h. Mortality of insects was observed. The insects were considered dead if appendages did not move when probed with a camel brush. The observed mortality data were corrected for control mortality using Abbott’s formula. Results from all replicates were subjected to probit analysis using the PriProbit Program V1.6.3 to determine LC_50_ values [19].

## Results and discussions

The yellow essential oil yield of *S. nivea* flowering aerial parts was 0.11% (V/W) and the density of the concentrated essential oil was determined as 0.81 g/ml. A total of 46 components of the essential oil were identified, accounting for 96.38% of the total oil. The principal compounds in the essential oil of *S. nivea* flowering aerial parts were (+)-limonene (15.46%), caryophyllene oxide (7.62%), linalool (7.20%), α-pinene (6.43%), β-pinene (5.66%) and spathulenol (5.02%) followed by β-eudesmol (4.64%) and eudesma-4,11-dien-2-ol (3.76%) (Table 1). Monoterpenoids represented 14 of the 43 compounds, corresponding to 45.96% of the whole oil while 23 of the 43 constituents were sesquiterpenoids (47.97% of the crude essential oil).

**Table 1:** **Chemical constituents of essential oil derived from* Saussurea nivea *flowering aerial part**

**Compounds**	RI*	**Peak Area (%)**
α-Pinene	939	6.43
β-Pinene	981	5.66
(+)-Limonene	1027	15.46
Benzeneacetaldehyde	1036	0.39
γ-Terpinene	1057	2.32
*cis*-Linalool oxide	1076	0.99
Linalool	1094	7.20
Phenylethyl Alcohol	1116	0.14
Nopinone	1142	0.48
Camphor	1146	0.56
Sabina ketone	1154	0.48
Borneol	1167	1.37
4-Terpineol	1175	1.05
*p*-Cymen-8-ol	1179	0.58
α-Terpineol	1188	1.77
Geraniol	1253	1.61
Nonanoic acid	1283	0.66
Chavibetol	1362	0.79
Copaene	1374	0.35
*trans*-β-Damascenone	1382	0.94
β-Bourbonene	1387	0.23
Dodecanal	1407	0.14
(*Z*)-Caryophyllene	1409	2.14
α-Cedrene	1411	0.13
Caryophyllene	1420	2.74
Germacrene D	1478	0.45
Geranyl acetone	1453	0.77
α-Caryophyllene	1454	1.85
γ-Muurolene	1473	0.79
α-Amorphene	1479	1.43
α-Curcumene	1483	1.34
β-Ionone	1487	2.09
α-Muurolene	1500	0.89
δ-Cadinene	1523	1.97
Dihydroactinolide	1538	2.07
α-Calacorene	1546	0.42
Spathulenol	1578	5.02
Caryophyllene oxide	1583	7.62
Isoaromadendrene epoxide	1594	1.19
Widdrol	1597	2.62
β-Eudesmol	1648	4.64
Eudesma-4,11-dien-2-ol	1691	3.76
γ-Costol	1732	2.87
Total		96.38
Monoterpenoids		45.96
Sesquiterpenoids		47.97
Others		2.47

*RI, retention index as determined on a HP-5MS column using the homologous series of *n*-hydrocarbons.

The essential oil of *S. nivea* flowering aerial parts exhibited contact toxicity against *S. zeamais* adults with an LD_50_ value of 10.56 μg/adult (Table 2). When compared with the positive control pyrethrum extract [20], the essential oil demonstrated 2.5 times less toxic against *S. zeamais*. However, compared with the other essential oils in the literature, the essential oil of *S. nivea* flowering aerial parts possessed stronger contact toxicity against *S. zeamais* adults, e.g. essential oils of *Artemisia lavandulaefoliaA. sieversianaA. capillariesA. mongolicaA. vestita* and *A. eriopoda* (LD_50_ = 55.2 μg/adult, 113.0 μg/adult, 106.0 μg/adult, 87.9 μg/adult, and 50.6 μg/adult, 24.8 μg/adult, respectively) [21-24], essential oil of *Schizonpeta multifida* (30.2 μg/adult) [25], essential oil of *Illicium simonsii* fruits (LD_50_ = 112.7 μg/adult) [26] and essential oil of *Cayratia japonica* (LD_50_ = 44.5 μg/adult) [27]. 

**Table 2:** **Contact toxicity (CT) and fumigant toxicity (FT) of* Saussurea nivea *essential oil against* Sitophilus zeamais *adults**

	**Treatment**	**LD**_**50**_**(μg/adult)**	**95% FL**	**Slope ± SE**	**Chi square (χ**^**2**^**)**
		**LC**_**50**_**(mg/L air)**			
CT	*S. nivea*	10.56	9.75-11.32	3.41 ± 0.35	16.22
	Pyrethrum extract*	4.29	3.86-4.72	-	-
FT	*S. nivea*	8.89	7.91-9.73	2.86 ± 0.30	13.37
	MeBr**	0.67	-	-	-

* from Wang et al. [20]. ** from Liu and Ho [1].

The essential oil of *S. nivea* flowering aerial parts possessed fumigant toxicity against the maize weevils with an LC_50_ value of 8.89 mg/L (Table 2). The commercial grain fumigant, methyl bromide (MeBr) was reported to have fumigant activity against *S. zeamais* adults with an LC_50_ value of 0.67 mg/L [1], thus the essential oil was 13 times less toxic to *S. zeamais* adults compared with MeBr. However, compared with fumigant activity of the other essential oils in the literature, the essential oil of *A. igniaria* exhibited stronger fumigant toxicity against *S. zeamais* adults, e.g. essential oils of *S. multifida*[25], *Kadsura heteroclite*[13]*, Murraya exotica*[28], and several essential oils from Genus *Artemisa*[21-24]. Moreover, one of the main constituent compounds, (+)-limonene has been commercialized for use as flea dips and shampoos for pets as well as sprays and aerosols [29] and was used to prepare for durable insect repellent cotton fabric [30]. It has been demonstrated to possess insecticidal activity against several stored-product insects such as the cowpea weevil (*Callosobruchus maculates*), lesser grain borer (*Rhyzopertha dominica*), flat grain beetle (*C. pusillus*), rice weevil (*S. oryzae*), maize weevil (*S. zeamais*), red flour beetle (*Tribolium castaneum*) and German cockroaches (*Blattella germanica*) [31-35]. Another main constituent compound, linalool was also found to have fumigant toxicity against the triatomine bug (*Rhodnius prolixus*) [36] and houseflies with a 24 h LC_50_ value of 13.6 mg/L [37]. Moreover, linalool possessed both contact and fumigant toxicity against human head louse (*Pediculus humanus capitis*) [38] and showed a high acaricidal activity by vapor action against mobile stages of *Tyrophagus putrescentiae*[39]. The two constituent compounds were demonstrated to be a potent inhibitor of acetylcholinesterase (AChE) activity from larvae of several stored product insects [34,40,41].

The above findings suggest that fumigant activity of the essential oil of *S. nivea* flowering aerial parts is quite promising by considering the currently used fumigants are synthetic insecticides and it shows potential to develop a possible new natural fumigant/insecticide for control of stored product insects. However, for the practical application of the essential oil as novel insecticide/fumigant, further studies on the safety of the essential oil to humans and on development of formulations are necessary to improve the efficacy and stability and to reduce cost.

## Conclusion

The study indicates that the essential oil of *S. nivea* flowering aerial parts has a potential for development into a new natural insecticide/fumigant for control of insects in stored grains. However, further studies on the safety of the oil in humans as well as development studies are required to optimize the efficacy and stability of this extract, and to reduce cost.

## Competing interest

The authors declare that they have no competing interests.

## Authors’ contributions

CSS carried out collection of plant sample, participated in bioassay, and performed the statistical analysis. JGH carried out GC and GC-MS, helped to draft the manuscript. LZL MT participated in the design of the study and bioassay, and drafted the manuscript. All the authors read and approved the final manuscript.
